# Activity of Medicinal Plant Extracts on Multiplication of* Mycobacterium tuberculosis* under Reduced Oxygen Conditions Using Intracellular and Axenic Assays

**DOI:** 10.1155/2016/8073079

**Published:** 2016-01-31

**Authors:** Purva D. Bhatter, Pooja D. Gupta, Tannaz J. Birdi

**Affiliations:** The Foundation for Medical Research, 84-A, RG Thadani Marg, Worli, Mumbai, Maharashtra 400 018, India

## Abstract

*Aim.* Test the activity of selected medicinal plant extracts on multiplication of* Mycobacterium tuberculosis* under reduced oxygen concentration which represents nonreplicating conditions.* Material and Methods.* Acetone, ethanol and aqueous extracts of the plants* Acorus calamus* L. (rhizome),* Ocimum sanctum* L. (leaf),* Piper nigrum* L. (seed), and* Pueraria tuberosa* DC. (tuber) were tested on* Mycobacterium tuberculosis* H37Rv intracellularly using an epithelial cell (A549) infection model. The extracts found to be active intracellularly were further studied axenically under reducing oxygen concentrations.* Results and Conclusions.* Intracellular multiplication was inhibited ≥60% by five of the twelve extracts. Amongst these 5 extracts, in axenic culture,* P. nigrum* (acetone) was active under aerobic, microaerophilic, and anaerobic conditions indicating presence of multiple components acting at different levels and* P. tuberosa* (aqueous) showed bactericidal activity under microaerophilic and anaerobic conditions implying the influence of anaerobiosis on its efficacy.* P. nigrum* (aqueous) and* A. calamus* (aqueous and ethanol) extracts were not active under axenic conditions but only inhibited intracellular growth of* Mycobacterium tuberculosis*, suggesting activation of host defense mechanisms to mediate bacterial killing rather than direct bactericidal activity.

## 1. Introduction

Globally, 8 million cases of tuberculosis (TB) are reported annually [1]. TB is characterized by actively dividing* Mycobacterium tuberculosis* (M.tb) in the alveolar macrophages and their dissemination through aerosols generated via cough/droplet nuclei.

Whilst axenic multiplication of M.tb requires aerobic conditions, a number of publications have reported that the bacterium can switch from aerobic/microaerophilic to anaerobic respiratory pathways [2] and may exist in three different respiratory states depending on the microenvironment encountered [3]. It has been postulated that M.tb under reduced oxygen conditions assumes dormancy in which it can remain viable for prolonged periods [4]. This dormancy is not a consequence of constant reactivation and destruction of the bacilli but rather of slower metabolism adopted to enter the stationary phase [5]. Despite quantitative metabolic shutdown dormant bacilli retain environmental responsiveness, indicating continuing metabolic integrity [6].

These nonreplicating, dormant bacilli represent a major reservoir for developing active TB. The current chemotherapeutic regimen mainly targets rapidly multiplying M.tb with the exception of pyrazinamide which is known to act on nonreplicating M.tb [7, 8].

Rapid acquisition of drug resistance in M.tb [9] demands alternative therapeutic approaches. Use of medicinal plants in TB treatment has been explored to strengthen chemotherapeutic regimens [10]. Most of these studies are targeted towards actively dividing bacteria and do not address the pool of “persisters.”

The current study aims to explore the efficacy of selected medicinal plants on slowly dividing M.tb. An assay system using alveolar epithelial cell line (A549) and an axenic anaerobic setup based on the modified Wayne method [11] was established to assess the activity of the plant extracts on H37Rv. Airway epithelial cells (AECs) harbor significantly higher numbers of intracellular bacteria and act as a preliminary defense against pathogen invasion by acting as a structural barrier. They also initiate and augment host airway defense through secretion of numerous antimicrobial agents, reactive oxygen species, nitric oxide, and proinflammatory chemokines and cytokines. The cytokines and chemokines secreted by AECs play an important role in attracting other cell types to the site of infection and thus assist in granuloma formation. Additionally in alveolar type II cells it has been reported that bacterial replication is higher compared to human or mouse macrophages* in vitro* [12, 13]. Thus we chose to explore an intracellular model based on type II AECs, represented by A549 cells.

## 2. Materials and Methods

### 2.1. Plant Material

*Acorus calamus, Ocimum sanctum, Piper nigrum,* and* Pueraria tuberosa* were selected on the basis of their broad antibacterial and immune-modulatory properties (Table 1). Plants were collected and authenticated by Dr. P. Tetali, Naoroji Godrej Centre for Plant Research (NGCPR). Voucher specimens were deposited at the Botanical Survey of India (BSI), Western Center, Pune, India, and NGCPR.

### 2.2. Extract Preparation

Coarsely powdered plant material was sequentially extracted [14] with acetone, ethanol, and distilled water using the Soxhlet apparatus. Respective solvent (300 mL) was continuously refluxed with 25 g of plant material for a period of 24–30 hours for efficient extraction of the phytoconstituents. After ethanol extraction and evaporation of the solvent, the aqueous extract was prepared by boiling the plant material until the volume of water was reduced to 25%. The aqueous extract was lyophilized (Thermo Fisher Scientific, USA) and the acetone and ethanol extracts were allowed to air-dry. The extracts were reconstituted at 20 mg/mL in dimethyl sulfoxide (DMSO), filtered through 0.2 *μ*m, 25 mm DMSO resistant Acrodisc syringe filters (Pall Corporation, USA), and stored at −20°C.

### 2.3. Bacterial Culture

The reference M.tb laboratory strain H37Rv was used. H37Rv was susceptible to the first-line drugs, isoniazid, ethambutol, rifampicin, and pyrazinamide.

### 2.4. Cytotoxicity Testing Using A549 Cell Line

The human lung carcinoma epithelial cell line, A549 (National Center for Cell Sciences, Pune, India), was used. Cells were grown in DMEM supplemented with 10% fetal calf serum (FCS) and 4 *μ*g/mL of gentamycin. The cytotoxicity testing was carried out by the neutral red uptake assay [15]. Briefly, 100 *μ*g/mL of the plant extract prepared in DMEM + 10% FCS was incubated overnight onto a 24 h culture of A549 cells and following incubation subjected to the neutral red assay. The assay was carried out in triplicate and repeated twice.

### 2.5. Intracellular Assay

A549 cells were grown in DMEM supplemented with 10% fetal calf serum (FCS) and 4 *μ*g/mL of gentamycin. 10^5^ cells/well were seeded in a 96-well plate and were infected with M.tb at a multiplicity of infection (MOI) of 1 : 1 for 6 h. After washing off the extracellular H37Rv, the cells were treated with amikacin (50 *μ*g/mL) for 2 h to kill the remaining extracellular bacteria. After amikacin treatment, the M.tb infected cells were incubated overnight with the plant extracts at a concentration of 25 *μ*g/mL, as no toxicity was observed on A549 cells with this concentration. On the following day the plant extract was washed off. On 0, 3rd, 5th, 7th, and 10th day after infection the cells were lysed with 0.1% sodium dodecyl sulphate to release the intracellular bacteria. The lysate was threefold serially diluted with PBS and 10 *μ*L of the highest two dilutions was spotted onto MiddleBrook 7H11 (MB7H11) agar plates supplemented with OADC (Becton Dickinson, USA) and 0.5% glycerol, the plates were incubated at 37°C for 3 weeks, and the CFUs (Colony Forming Units) were enumerated.

### 2.6. Axenic Assay under Differential Oxygen Concentration

MiddleBrook 7H9 broth (10 mL/tube), supplemented with ADC and 0.5% glycerol, was aliquoted and the bacterial suspension containing 10^4^ CFUs/mL was inoculated into the tubes. Since our earlier study had shown that these plant extracts were inactive at lower concentrations under aerobic axenic conditions [16] 100 *μ*g/mL concentration of the plant extracts was used. Positive (viable M.tb, VMTB) and medium (MB7H9) controls along with a rifampicin (1 *μ*g/mL) control were maintained. The above setup in triplicate was subjected to differentially reducing oxygen concentration, namely, aerobic, microaerophilic, and anaerobic conditions. The microaerophilic conditions were obtained using the candle jar method [17]. The anaerobic conditions were achieved in an anaerobic jar with a gas pack (HiMedia, India) and confirmed using the indicator tablets provided by the manufacturer. The sets were incubated for a period of 10 days. After incubation all of the tubes were vortex mixed and serially diluted 10-fold and 10 *μ*L of this dilution was spotted on MB7H11 agar plate supplemented with OADC and 0.5% glycerol. The plates were incubated at 37°C and under aerobic conditions to ensure growth of bacteria.

## 3. Results

### 3.1. Effect of Plant Extracts on Intracellular Growth of H37Rv

**Figure 1:**
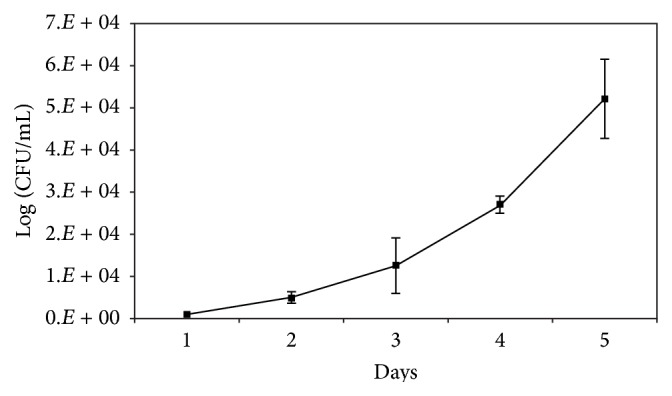
Intracellular growth of H37Rv over a period of 10 days in A549 based epithelial cell model. The results are represented as mean ± SD of three experiments. An increase of ~1.5 log is observed in growth.

**Figure 2:**
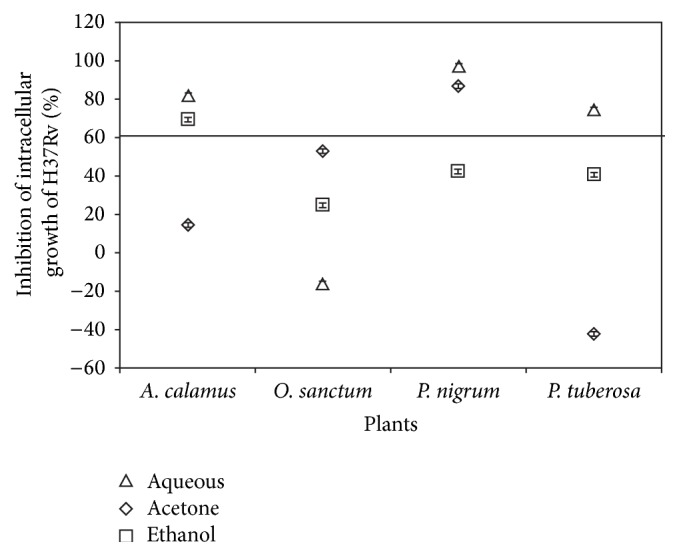
Intracellular inhibition of growth of H37Rv. Extracts of 4 plants were tested on intracellular growth of H37Rv in A549 cells at 25 *μ*g/mL with MOI of 1 : 1. The extracts showing greater than 60% inhibition of bacterial growth 10 days after infection were selected for testing under anaerobic conditions in an axenic setup.

The plant extracts were assessed at 100 *μ*g/mL for their toxicity in an* in vitro* assay system using A549 cells and were found to be nontoxic (Table 2). The growth of H37Rv in an intracellular model using A549 is demonstrated in Figure 1. Twelve plant extracts (25 *μ*g/mL) were tested for their ability to inhibit H37Rv intracellularly. Greater than 90% inhibition of bacterial growth was observed only with* P. nigrum* aqueous and acetone extracts. Other plant extracts showed inhibition of bacterial growth ranging from 16% to 86% (Figure 2).

Five plant extracts which showed more than 60% inhibition of intracellular bacterial growth (Figure 2) were considered active. The active extracts were tested further for their activity in axenic culture under anaerobic conditions to ascertain whether their activity was due to their ability to stimulate the macrophages or if their bactericidal properties were expressed under anaerobic conditions.

### 3.2. Effect of Plant Extracts on Growth of H37Rv under Reducing Oxygen Conditions

**Figure 3:**
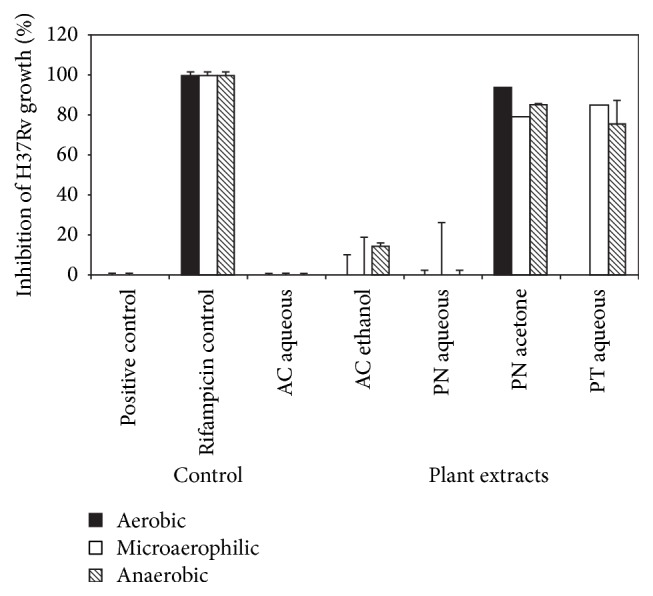
Activity of plant extracts under reducing oxygen concentration against H37Rv. The 5 intracellularly active plant extracts were tested on H37Rv under reducing oxygen concentration. Viable M.tb acted as the positive control whereas rifampicin was used as the experimental control.* P. nigrum* acetone extracts showed inhibition of bacterial growth under all 3 conditions. The results are represented as mean ± SD of three experiments.

It was observed that the generation time of H37Rv under aerobic, microaerophilic, and anaerobic conditions was 36.12 ± 0.6 h, 40.63 ± 0.9 h, and 45.1 ± 0.3 h, respectively, implying compromised multiplication of M.tb under reducing oxygen conditions. Under reducing oxygen concentrations, 2/5 plant extracts showed >50% inhibition of growth (Figure 3).* P. nigrum* acetone extract was effective under all 3 conditions of growth, namely, aerobic (*p* = 0.033), microaerophilic (*p* = 0.004), and anaerobic (*p* = 0.046).* P. tuberosa* aqueous extract showed bactericidal activity under microaerophilic (*p* = 0.015) and anaerobic (*p* = 0.096) conditions. The remaining three extracts were not active (Figure 3).

## 4. Discussion

Due to increasing drug resistance, alternative approaches for treatment of TB and MDR TB are being sought.

There is ample clinical and animal experimental evidence that M.tb can persist in tissues for months to decades without replicating and resumes growth to cause active TB [18]. One-third of the population in TB endemic regions acts as reservoirs of these dormant, nonreplicating bacilli [19]. While previous studies [10, 20] focus on activity against actively dividing bacteria, very few report efficacy against the nonreplicating bacteria. Hence it is important to study the efficacy of plant extracts on the latter.

It has been observed that when pure isolated compounds are tested individually at a concentration at which they occur in a plant, they are often less active than the plant itself implying that crude plant extracts may be exerting their activity through synergistic/additive action [21–26]. Thus the present study preferred crude extracts over individual active components.

*A. calamus* (aqueous, ethanol) and* P. nigrum* (aqueous) were unable to inhibit the growth of H37Rv under axenic condition. However, in the presence of these extracts, intracellular growth was abrogated. The most likely reason would be that the extracts activated the host cell to kill the bacteria. Nevertheless, it cannot be ruled out that the extracts could be modified/altered by the host cell to exhibit bactericidal action.

On the other hand efficacy of* P. tuberosa* (aqueous) under intracellular, microaerophilic, and anaerobic conditions but not under axenic aerobic conditions may be indicative of its activity only under reduced oxygen concentration. It has been suggested that low oxygen levels can enhance the activity of essential oils present in plant extracts. Diminished oxygen supply leads to fewer oxidative changes in the essential oils and/or the fact that cells obtaining energy via anaerobic metabolism are more sensitive to the toxic action of essential oils [27].

*P. nigrum* (acetone) was active both intracellularly and in axenic cultures under varying oxygen conditions (aerobic, microaerophilic, and anaerobic) suggesting either a single mode of action that affects multiple bacterial physiological states or the presence of multiple components acting at different levels.

The active extracts identified through this preliminary study with the standard laboratory strain H37Rv need to be screened on clinical isolates. If confirmed, the potential of adjunct therapy of TB with such extracts is viable.

## Figures and Tables

**Table 1:** Selection criteria for plants and their percentage yield.

Sr. number	Botanical name (Herbarium number)	Acronym	Common name	Area of collection	Parts used	Selection criteria^*∗*^	Percent yield^#^		
							Acetone	Ethanol	Aqueous
1	*Acorus calamus *Linn. (BSI-131716)	AC	Sweet flag	Shindewadi, Satara district, Maharashtra, India	Rhizome	Antibacterial, immune-modulatory	10	7.04	10.8

2	*Ocimum sanctum* Linn. (BSI-131712)	OS	Indian tulsi	Shindewadi, Satara district, Maharashtra, India	Leaves	Immune-modulatory	6.0	3.62	24.0

3	*Piper nigrum* Linn. (BSI-131714)	PN	Black pepper	Kottayam, Kerala, India	Seed	Antibacterial	3.16	6.64	22.64

4	*Pueraria tuberosa *DC. (NGCPR-670)	PT	Indian kudzu	Mulshi, Pune district, Maharashtra, India	Rhizome	Immune-modulatory	6.72	5.92	22.83

^*∗*^The plants were selected for the present study based on their biological activities mentioned under the column “selection criteria.”

^#^Percentage yield w/w after sequential extraction using acetone, ethanol, and aqueous extracts.

**Table 2:** Cytotoxicity of the four plants used in intracellular assay using A549 cells.

Plant	Extract (100 *µ*g/mL)	% viability of A549 cells^*∗*^
*A. calamus*	Acetone	96 ± 3
	Ethanol	113 ± 7
	Aqueous	102 ± 32

*O. sanctum*	Acetone	97 ± 5
	Ethanol	73 ± 14
	Aqueous	114 ± 19

*P. nigrum*	Acetone	90 ± 7
	Ethanol	98 ± 19
	Aqueous	118 ± 11

*P. tuberosa*	Acetone	118 ± 20
	Ethanol	97 ± 19
	Aqueous	82 ± 14

^*∗*^Values represent mean ± SD of % viable A549 cells from two independent assays.
